# The Occurrence and Characteristics of Methicillin-Resistant Staphylococcal Isolates from Foods and Containers

**DOI:** 10.3390/antibiotics12081287

**Published:** 2023-08-04

**Authors:** Rada Kansaen, Parichart Boueroy, Rujirat Hatrongjit, Watcharaporn Kamjumphol, Anusak Kerdsin, Peechanika Chopjitt

**Affiliations:** 1 Faculty of Public Health, Chalermphrakiat Sakon Nakhon Province Campus, Kasetsart University, Sakon Nakhon 47000, Thailand; rada.kan@ku.th (R.K.); parichart.bou@ku.th (P.B.); anusak.ke@ku.th (A.K.); 2 Faculty of Science and Engineering, Chalermphrakiat Sakon Nakhon Province Campus, Kasetsart University, Sakon Nakhon 47000, Thailand; rujirat.ha@ku.th; 3 National Institute of Health, Department of Medical Sciences, Ministry of Public Health, Nonthaburi 11000, Thailand; watcharaporn.k@dmsc.mail.go.th

**Keywords:** foods, methicillin-resistant *Staphylococcus* spp., SCC*mec* type, multilocus sequencing typing, *spa* type, staphylococcal enterotoxins (SEs)

## Abstract

Antimicrobial resistance (AMR) has emerged as an urgent global public health issue that requires immediate attention. Methicillin-resistant staphylococci (MRS) is a major problem, as it may cause serious human and animal infections, eventually resulting in death. This study determined the proportional distribution, genetic characteristics, and antimicrobial susceptibility of *mec*A- or *mec*C-carrying staphylococci isolated from food chain products. A total of 230 samples were taken from meat, food, fermented food, and food containers. Overall, 13.9% (32/230) of the samples were identified to have *Staphylococcus aureus* isolates; of those, 3.9% (9/230) were MRS, with eight mecA-positive and one mecC-positive samples, and 1.3% (3/230) methicillin-resistant *Staphylococcus aureus* (MRSA). MRSA strains belonging to three sequence types (ST9, ST22, and a newly identified ST), three different *spa* types (T005, t526, and a newly identified type), and three different SCC*mec* types (IV, V, and an unidentified SCC*mec*) were detected. Additionally, eight *mec*A-positive staphylococcal isolates were identified as *S. haemolyticus*, *S. sciuri*, *S. simulans*, and *S. warneri*, while the *mec*C-harboring isolate was *S. xylosus*. The enterotoxin gene, *SEm*, was detected at 1.56% in *S. aureus*, whereas *SEq* was detected at 0.31%, and *SEi* was also found in MRSA. Our study emphasizes the importance of enhanced hygiene standards in reducing the risk of occupational and foodborne MRSA infections associated with the handling or consumption of meat, food, and preserved food products.

## 1. Introduction

Staphylococci are natural inhabitants of the skin and mucous membranes in both humans and various animals. They are typically classified into two groups based on their ability to produce coagulase: coagulase-positive (CoPS) and coagulase-negative staphylococci (CoNS) [1]. *Staphylococcus aureus* (*S. aureus*), a CoPS member, is widely recognized as a major causative agent of food poisoning and infections in both clinical and community settings [2,3,4,5]. The production of coagulase by *S. aureus* promotes blood clotting, and the resulting fibrin coat on the bacterial surface may facilitate the evasion of the immune system. CoNS consist of numerous species, including opportunistic pathogens such as *S. epidermidis*, *S. capitis*, *S. hominis*, *S. haemolyticus*, *S. saccharolyticus*, *S. warneri*, *S. lugdunensis*, *S. saprophyticus*, and *S. cohnii*. Although CoNS lack the ability to produce coagulase, they possess species and strain-specific virulence factors that contribute to their role as notorious opportunistic pathogens. One significant pathogenicity mechanism employed by CoNS is their ability to form biofilms, allowing them to colonize both abiotic surfaces of medical devices and biotic surfaces such as host tissues coated with host factors [6].

This opportunistic pathogen is capable of infecting both humans and other mammals, resulting in a broad spectrum of diseases. These include food poisoning, which manifests as abdominal pains, diarrhea, nausea, and vomiting, as well as more serious conditions such as endocarditis, pneumonia, osteomyelitis, toxic shock syndrome, septicemia, and soft tissue and skin infections [7,8]. In addition, *S. aureus* is frequently found in animal-derived foods such as undercooked meat and dairy products [9,10,11]. Its ability to survive in a variety of environments and to cause such a wide range of diseases highlights the need for effective prevention and control measures concerning public health.

The pathogenicity of *S. aureus* is attributed to a combination of factors that contribute to its invasive nature, the production of extracellular factors, and its antibiotic resistance. In order to enhance the process of pathogenesis and facilitate udder infection, *S. aureus* has developed a range of virulence factors. These factors include various extracellular enzymes such as lipases, proteases, amylases, hyaluronidase, DNases, coagulase, lactamase, hemolysins, and capsules [12]. Additionally, *S. aureus* produces enterotoxins (SEs: SEA to SEE) and non-classical SE-like toxins (SEl: SEG to SEU) that are associated with food poisoning. Notably, these toxins are resistant to heat, proteolytic enzymes, and low pH conditions. Furthermore, *S. aureus* is known to produce toxic shock syndrome toxin 1 (TSST-1), a potent superantigenic toxin. The presence of TSST-1 can lead to severe symptoms such as high fever, rash, shock syndrome, hypotension, and the inflammation of the blood system, as well as to the Panton–Valentine leucocidin (PVL), which causes leukocytosis along with necrosis on the skin or mucosa surface, and TSST-1 is capable of inducing lysis of human neutrophils and enhances the adherence of *S. aureus* to the extracellular matrix [12,13,14,15].

The emergence of methicillin-resistant staphylococci (MRS) presents a significant and concerning threat, as these strains exhibit resistance to all beta-lactam antibiotics, thereby compromising treatment options and increasing the risk of life-threatening infections. It is important to recognize that other coagulase-positive (*S. aureus*, *S. schleiferi*, *S. delphini*, *S. intermedius*, *S pseudintermedius*, and *S. lutrae*) and coagulase-negative MRS species (*S. cohnii*, *S. epidermidis*, *S. haemolyticus*, *S. hominis*, *S. lentus*, *S. lugdunensis*, *S. sciuri*, and *S. xylosus*) have gained significance in recent years. These species have been implicated in a variety of opportunistic infections, particularly among immunocompromised patients [16]. The development of methicillin resistance is primarily attributed to the presence of the *mec*A gene, a pivotal genetic element located on the mobile genetic element known as the staphylococcal cassette chromosome mec (SCC*mec*). This genetic element encodes an altered penicillin-binding protein (PBP2a), which imparts resistance to methicillin and other beta-lactam antibiotics. In addition to *mec*A, the presence of other *mec* genes, including *mec*B and *mec*C, has also been recognized as being associated with beta-lactam resistance [1]. This gene is widespread in *S. aureus* and coagulase-negative staphylococci (CoNS) from both human and animal origin [17,18]. The widespread consumption of antibiotics in the livestock sector has led to their persistent release into the environment and increased antibiotic-resistant bacteria. Numerous studies have demonstrated the presence of *mec*A-positive methicillin-resistant *Staphylococcus aureus* (MRSA) in various food sources, such as retail meat, fish, poultry, pork, beef, ready-to-eat foods, and even vegetables [11,19,20,21]. Additionally, CoNS carrying *mec*A, which are known for their increasing rates of methicillin resistance, have been detected in milk at a rate of 0.6% in Brazil and 6.7% in Tunisia [22,23], in ready-to-eat foods at a rate of 16.4% in Poland [24], and in meat at a rate 2.3–8% in Egypt [25,26], which raises additional concerns about the spread of resistance. In Thailand, the prevalence of MRA was found to be 20.5% in the university environment and 52.3% in the hospital environment [27]. The prevalence of MRSA in meat has been reported as 44.8–50% [28,29]. However, a lower prevalence of MRSA (3.8%) in non-human isolates was reported [30].

The presence of staphylococci in meat is oftentimes because personnel participating in the production process engage in unhygienic behaviors during the processing, shipping, slicing, storage, and point-of-sale stages throughout the production process. By evaluating these factors, valuable insights into the potential transmission of antibiotic resistance and virulence factors through the food chain can be obtained. In light of these findings, the purpose of the present study was to investigate the distribution of methicillin-resistant staphylococci in various food types, with a concentration on characterizing their SCC*mec* types, spa types, and the presence of enterotoxin genes. 

## 2. Results 

### 2.1. Distribution of Methicillin-Resistant Staphylococci 

From the 230 samples, 666 staphylococcal isolates were identified comprising 3 MRSA isolates from 3 samples (1 pork and 2 beef; 3/230, 1.30%); 8 MRS carrying *mec*A from 8 samples (8/230, 3.47%) consisting of pork (*n* = 4), beef (*n* = 2), and chicken (*n* = 2); 1 MRS harboring *mec*C from pork (1/230, 0.43%); and 70 *S. aureus* (methicillin-susceptible) isolates from 32 samples (32/230, 13.91%) consisting of pork (*n* = 6), beef (*n* = 5), chicken (*n* = 19), and fermented food (*n* = 2). (Table 1) 

The *mec*A-harboring MRS (n = 8) were identified as three *S. haemolyticus* strains, two *S. sciuri* strains, two *S. warneri* strains, and one *S. stimulans* strain. We identified one MRS harboring *mec*C as *S. xylosus* (n = 1). These are summarized in Table 2. The proportion of methicillin-resistant staphylococci (MRSA and MRS) present in foods was 12/320, 5.21% in the current study.

**Figure 1:**
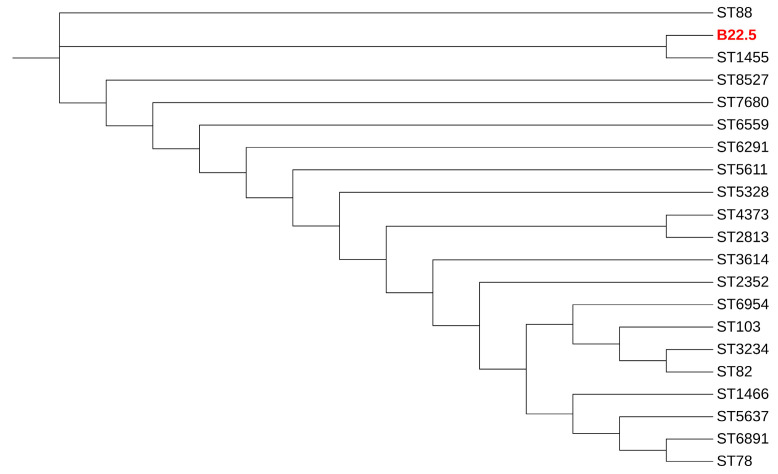
Dendrogram of concatenated sequences of seven MLST loci of MRSA strain B22.5 (red color), as a new ST isolate, and its related STs.

As shown in Table 2, MRSA strain no. B(3)1.2 belonged to SCC*mec* type IV, ST22, *spa* t005, and carried the pvl gene. MRSA strain no. P(2)12.4 showed unidentified SCC*mec* types, ST9, and *spa* t526, whereas MRSA strain no. B22.5 was SCC*mec* type V, a new *spa* type and a new ST; however, it is closely related to ST1455, which is isolated from the lung aspirate of a Chinese patient, as shown in Figure 1. Among the eight *mec*A-harboring MRS isolates, the most common SCC*mec* type was an unidentified SCCmec type (5/8, 62.5%), followed by SCCmec types V (2/8, 25%) and III (1/8, 12.5%), as shown in Table 2. Finally, the *mec*C-harboring *S. xylosus* carried an unidentified SCC*mec* type (Table 2).

### 2.2. Antimicrobial Resistance

All three of the MRSA isolates were multidrug-resistant (MDR), with resistance to erythromycin, oxacillin, cefoxitin, gentamycin, azithromycin, and ciprofloxacin, while two isolates (strain no. P(2)12.4 and B(3)1.2) could induce clindamycin resistance. The *mec*A- and *mec*C-carrying staphylococci were classified as MDR in seven strains except for *S. haemolyticus* strain no.C49.2 and *S. xylosus* strain no P20.3, as shown in Table 2. However, all isolates were susceptible to vancomycin, linezolid, and rifampin. 

### 2.3. Detection of Foodborne Staphylococcus Aureus Enterotoxin Genes

In total, 73 *S. aureus* isolates, including 3 MRSA, were detected with five enterotoxin genes (*SEj*, *SEl*, *Seq*, *Sem*, and *SEr*). The *SEm* gene was found in *S. aureus* (5/73, 6.85%), and the *SEq* and *SEj* genes were found in MRSA strain no B22.5 (1/73, 1.37%).

## 3. Discussion

Antibiotic resistance bacteria are a major global health problem, emerging in a variety of environmental samples. To better comprehend the dissemination of methicillin-resistant staphylococci in food product chains in northeast Thailand, we characterized staphylococcal isolates from foods, food containers, and meat. Our study showed that the proportion of *S. aureus* (10%) was lower than in other studies in Thailand, for example, 83% (87/105) of *S. aureus* present in ready-to-eat food samples in Songkhla Province [31] and 60% (36/60) in fermented pork sausage in Amnatcharoen Province [32]. In other countries, *S. aureus* has been found in retail raw meat samples: 21.23% (96/452) in Tukey [33], 21.81% (89/408) in India [34], 16.9–35% in China [35,36,37], 33.9% (165/487) in Chile [38], and 13.8% (22/160) in Greece [39]. In contrast, in the current study, the proportion of MRSA in food samples was also low (0.94%; 3/320), which was less than for the proportions reported in other regions of Thailand, such as 20% (2/10) [28] and 44.8% (55/116) [29], both in retail pork samples. However, some studies showed a low prevalence of MRSA in non-human samples, for example, 2.2% from retail food and food handlers’ gloves, 1.7% in beef, 1.2–1.9% in pork, 0.3% in chicken, 3.5% in turkey, 1.86% in secondary school environments, and 1.58% from environmental contamination in railway stations and coach stations [30]. Differences in the sampling period, sample size, sampling site, sampling techniques, isolation method, single enrichment step, the frequency of MRSA in different samples, or geographical locations could partially explain the variation in prevalence. However, these results highlight the necessity to mitigate the risk of *S. aureus* and MRSA transmission via meat products to humans in the food supply chain. 

The current study revealed that SCC*mec* types IV and V were detected concordant with several studies in retail meat products worldwide [40,41,42,43,44]. One MRSA in the current study was ST9, which is predominant in most Asian countries, including Taiwan, Hong Kong, Malaysia, and Thailand [45,46,47,48,49]. The ST9 strains are generally MDR, with >80% resistance to erythromycin, ciprofloxacin, gentamicin, tetracycline, and clindamycin [50], which was similar to our strain in the current study. Our MRSA ST22 strain is the epidemic clone EMRSA-15, and it is a hospital-associated pathogen, typically resistant to ciprofloxacin and erythromycin [51,52,53]. However, some studies have reported MRSA ST22 isolated from animals [54,55]. It is interesting that a novel ST of MRSA was identified from beef samples in the current study. This ST was closely related to ST 1455, which was isolated from a human patient’s bronchoalveolar lavage [56]. Therefore, this novel ST should be subjected to monitoring and surveillance.

The *mec*A-carrying staphylococcal isolates other than MRSA in the current study belonged to five species of coagulase-negative staphylococci, namely *S. haemolyticus* (37.5%), *S. sciuri* (25%), *S. warneri* (25%), and *S. simulans* (12.5%), while the *mec*C-harboring isolate was *S. xylosus*. In Egypt, Osman et al. detected *S. hyicus* (30%), *S. intermedius* (15%), *S. epidermidis* (5%), *S. hemilyticus* (5%), *S. hominis* (5%), *S. lugdumenis* (15%), *S. simulants* (5%), and *S. scuri* (20%) in imported beef meat [25]. Boamah et al. identified *S. gallinarum* (32%); *S*. *saprophyticus* (20%); *S. chromogens* (20%); *S. warneri* (12%); *S. hominis* (8%); *S. caprae* and *S. epidermidis* (4%); *S. sciuri* (42.97%); *S. lentus* (35.94%); *S. xylosus* (4.30%); *S. haemolyticus* (3.91%); *S. saprophyticus* (1.95%); and *S. cohnii* (0.39%) in poultry in Ghana [57]. Pimenta et al. found *S. gallinarum* (35.2%); *S. simulans* (17%); *S. sciuri* (10.2%); *S. lentus* (4.5%); and *S. cohnii* and *S. xylosus* (2.2%) in broiler chicken products in Brazil [58]. Moreover, in Korea, *S. agnetis* (19.4%), *S. saprophyticus* (19%), *S. chromogens* (14.5%), S *hyicus* (12.9%), and *S. sciuri* (13.8%) were detected in retail chicken meat [1]. These findings suggest that the frequent occurrence of non-aureus staphylococci in meat may be a hazard associated with food and public health safety. Some of them can cause foodborne infections [59,60], contribute to antibiotic resistance transmission [61], and lead to zoonotic infections [62]. Therefore, close monitoring should be carefully considered.

Regarding the risk of foodborne intoxication, numerous surveys of staphylococcal enterotoxins (SEs) have been reported, which have identified five classical enterotoxin types, SEa to SEe [63], and many new types of SEs have been reported: SEg, SEh, SEi, SEk, SEl, SEm, SEn, SEo, SEp, SEq, SEr, and SEu [64]. One of the limitations of this study is that we did not detect the classical enterotoxin genes; therefore, these classical enterotoxin genes could not be ruled out in our *S. aureus* isolates. Hu et al. showed five new types of enterotoxin genes, namely SEj, SEl, SEq, SEm, and SEr. Of these, SEj and SEr were detected in 16.6% and 14.3%, respectively [65]. Additionally, SEi (97.2%) and SEm (86.1%) were frequently detected in retail foods in China [11]. In contrast, our study revealed SEm in *S. aureus* (6.1%, 5/82) and SEq and SEi in MRSA (1.2%, 1/82). There has been a rise in the number of foodborne staphylococcal isolates, especially MRSA, which is linked to novel enterotoxins; therefore, these data indicate that we should pay attention to both types of toxins. In addition, the five new enterotoxin genes were extensively present in proteins of animal origin compared with that from other origins. This is related to the animal characteristics and interaction with the living environment, operation environment for food processing, and storage environment for finished products [65]. 

## 4. Materials and Methods

### 4.1. Ethical Statement 

Ethical review and approval were not required because this study did not involve human subjects.

### 4.2. Sample Collection, Isolation, and Presumptive Deification 

From June to December 2019, a total of 230 samples were taken from various foods and storage containers located in rural northeastern Thailand. A variety of samples, including food containers, meat, pork, chicken, beef, and pickled food, were gathered. The samples included 80 food container samples, 30 food samples, 90 meat samples (30 pork, 30 chicken, and 30 beef), and 30 pickled food samples. The collection of samples was performed in sterile conditions. Storage containers were swabbed using sterile cotton that was immediately placed in 1 mL of Mannitol salt broth (MSB) (HiMedia Laboratories Pvt. Ltd.; Nashik, India). Food product samples were collected in accordance with Sorour et al. [26]. The samples were transferred to the laboratory in sterile plastic bags. A 10 g amount of each food sample was diluted with 90 mL of buffer peptone water (BPW) (HiMedia Laboratories Pvt. Ltd.; Nashik, India), incubated overnight at 37 °C under aerobic conditions, and then streaked on mannitol salt agar medium (MSA) (HiMedia Laboratories Pvt. Ltd.; Nashik, India) before incubating at 37 °C and examined after 24 h to 48 h. There was a presumption that the colonies on MSA, which were colored yellow and pink, were staphylococci. Following the preliminary fundamental phenotypic examination (which included a microscopic inspection, Gram staining, catalase production, and coagulase tube test utilizing rabbit plasma), these isolates were identified at the species level via either PCR or DNA sequencing, as will be detailed in subsequent sections.

### 4.3. Microbiology and Molecular Characterization of S. Aureus and MRSA 

In accordance with the protocol provided by the manufacturer, total genomic DNA was extracted using a ZymoBIOMICsTM DNA Miniprep Kit (Zymo Research; Irvine, CA, USA). The quantity and purity of DNA were determined using a NanoDropTM 2000 Spectrometer (Thermo Fisher Scientific; Waltham, MA, USA), and the DNA sample was stored at −20 °C for further study.

Multiplex PCR was performed to detect *fem*A genes specific for *S. aureus* species, and the *mec*A, *mec*C, and *luk*S genes following a previously established protocol [66,67]. The sequence primers are shown in Table 3. DNA amplification was carried out in 25 μL of a PCR mixture that contained 12.5 μL of 2x JumpStart™ REDTaq^®^ ReadyMix™ Reaction Mix (SIGMA; Saint Louis, MO, USA), 0.4 μM of each primer, 100 ng of the DNA sample, and sterile deionized water. PCR was carried out using the following thermal cyclic conditions: initial denaturation at 94 °C for 3 min, followed by 35 cycles of denaturation at 94 °C for 30 s, annealing at 55 °C for 30 s, an extension at 72 °C for 30 s, a final extension of 72 °C for 5 min, and cooling to 4 °C. 

### 4.4. Enterotoxin Genes and PVL Detection 

*S. aureus* isolates were subjected to PCR for the identification of five enterotoxin genes, namely *SEj*, *SEl*, *SEq*, *SEm*, and *SEr*, as described elsewhere [65]. Briefly, the total reaction volume was 25 μL and included the following: 12.5 μL 2x Mytaq^TM^ HS Red Mix (Bioline Reagents Ltd.; London, UK), sterile deionized water, 1 μM of each primer, and 100 ng DNA template. The PCR conditions were as follows: pre-denaturation at 94 °C, 40 s; annealing at 52 °C, 40 s; and extension at 72 °C, 1 min for a total of 35 cycles; and final extension for at 72 °C, 10 min. This procedure was used for all genes except *SEj*, for which the annealing temperature was 55 °C. 

### 4.5. Sequencing of mecA-or mecC-Harboring Staphylococci

Sequencing was carried out as described by Poyart et al. [68]. The DNA samples were amplified for the *sod*A gene with the primer sodA-F (5′ CCITAYICITAYGAYGCIYTIGARCC-3′) and sodA-R (5′-ARRTARTAIGCRT GYTCCCAIACRTC-3′). Briefly, 50 µL of the reaction mixture was used, which contained 25 µL of 2x Mytaq^TM^ HS Red Mix (Bioline Reagents Ltd.; London, UK), sterile deionized water, 0.75 µM of each primer, and 100 ng of bacterial DNA sample. Thermal cycling reaction conditions consisted of initial denaturing at 95 °C for 3 min and then being subjected to 35 cycles of amplification, denaturation at 95 °C for 30 s, annealing at 37 °C for 60 s, and elongation at 72 °C for 45 s. The PCR amplicons were purified using a GF-1 AmbiClean Kit (Vivantis Technologies Sdn Bhd; Kuala Lumpur, Malaysia) and then sequenced at 1st BASE products and services company, Malaysia. The Basic Local Alignment Search Tool (BLASTN) was used to identify species of staphylococci using a cut-off value of ≥97% [69].

### 4.6. Molecular Typing

To determine the Staphylococcal Chromosomal Cassette (SCC*mec*) type, a multiplex PCR (M-PCR) was performed according to the method described by Kondo et al. [68]. M-PCR 1, designed for the *ccr* type assignment, employed two primers for *mec*A detection and eight primers for the identification of five *ccr* genes. Within these eight primers, there were four primers that included a forward primer shared by *ccrB1-3* and three reverse primers specific to *ccrA1*, *ccrA2*, and *ccrA3*. This allowed for the identification of *ccr1-3* based on the differences in the *ccrA* genes. Additionally, two primers were utilized for identifying *ccr4* and two for identifying *ccr5*. In M-PCR 2, which aimed to assign *mec* classes, four primers were employed to identify the gene lineages of *mec*A-*mec*I (class A *mec*), *mec*A-IS1272 (class B *mec*), and *mec*A-IS431 (class C *mec*). The PCR reaction mixture for both M-PCR 1 and M-PCR 2 consisted of 100 ng of DNA extract in a total volume of 25 µL. This mixture included 12.5 μlx of 2× JumpStart™ REDTaq^®^ ReadyMix™ PCR Reaction Mix (SIGMA; Saint Louis, MO, USA) and a concentration of 0.2 µM for each primer. The thermal cycling conditions involved an initial denaturation step at 94 °C for 2 min, followed by 35 cycles of denaturation at 94 °C for 2 min, annealing at 57 °C for 1 min, and extension at 72 °C for 2 min. The amplification process concluded with a final extension step at 72 °C for 2 min. 

Multilocus sequence typing (MLST) was performed following the protocol described elsewhere [70]. Seven housekeeping genes (*arcC*, *aroE*, *glpF*, *gmk*, *pta*, *tpi*, and *yqiL*) were amplified using PCR. The PCRs were carried out with 50 μL reaction volumes containing 12.5 μL 2x Mytaq^TM^ HS Red Mix (Bioline Reagents Ltd.; London, UK), 2.5 µM of each primer, 100 ng bacterial DNA sample, and sterile deionized water. PCR amplification was performed with thermal cycling reaction conditions consisting of initial denaturation at 95 °C for 5 min, followed by 35 cycles of denaturation at 95 °C for 1 min, annealing at 55 °C for 1 min, and extension at 72 °C for 1 min, and the cycle was completed with a single extension at 72 °C for 5 min. The PCR amplicons were purified using a GF-1 AmbiClean Kit (Vivantis Technologies Sdn Bhd; Kuala Lumpur, Malaysia) and then sequenced at 1st BASE products and services company, Malaysia. The alleles and sequence types (STs) were identified using the scheme published in multilocus sequence typing databases (https://pubmlst.org/organisms/staphylococcus-aureus, (accessed on 20 February 2023). 

The *spa* typing was performed via the amplification of polymorphic X region of the *S. aureus* protein A gene (*spa*) using the standard primers spa-1095F (5′-AGACGATCCTTCGGTGAGC3′) and spa-1517R (5′-GCTTTTGCAATGTCATTTACTG3′) and a PCR program described elsewhere [71]. Briefly, 50 µL of the reaction mixture was used, which contained 25 μL 2X Mytaq^TM^ HS Red Mix (Bioline Reagents Ltd., London, UK), sterile deionized water, and 100 ng of the bacterial DNA sample. Thermal cycling reaction conditions consisted of initial denaturation at 80 °C, 5 min; 35 cycles of denaturation at 94 °C, 45 s; annealing at 60 °C, 45 s; and extension at 72 °C, 90 s; and finally, a single extension at 72 °C, 10 min. The PCR amplicons were purified using a GF-1 AmbiClean Kit (Vivantis Technologies Sdn Bhd; Kuala Lumpur, Malaysia) and then sequenced at 1st BASE products and services company, Malaysia. Spa types were determined with the *Spa* Typer website http://spatyper.fortinbras.us, (accessed on 20 February 2023).

### 4.7. Electrophoretic Analysis of PCR Products

After amplification, 5 μL of PCR product was subjected to analysis on 2% agarose gel (Bioline Reagents Ltd., London, UK) in 0.5X TBE buffer (Omega BioTek, Inc; Norcross, Georgia) to determine the molecular weight of the amplified DNA fragment. The 5 μL GeneRuler 100 bp Plus DNA ladder (Thermo Scientific; Vilnius, Lithuania) was loaded onto the same agarose gel as a molecular weight standard. Subsequently, the gel was stained with ethidium bromide (Wako, Wako Pure Chemical Industries, Ltd.; Tokyo, Japan) and destained by soaking it in water. Electrophoresis was performed on horizontal electrophoresis equipment (Mupid-Exu; Chuo-ku, Japan) for 30 min at a constant 100 Volts. Subsequently, the gel was visualized using a UV Transilluminator (SynGene; Cambridge, UK), enabling a comparison between the migration patterns of the DNA ladder bands and the PCR products. 

### 4.8. Analysis of New STs

The construction of the phylogenetic tree for STs that are closely related to strain B22.5, a new ST, was performed in this study via Phylogeny.fr [72]. The phylogenetic tree was visualized using the Interactive Tree of Life (iTOL) (http://itol.embl.de, (accessed on 7 July 2023) [73].

### 4.9. Antimicrobial Susceptibility Testing

The antimicrobial susceptibility test was performed using disk diffusion in Mueller–Hinton agar (Merck; Darmstadt, Germany) according to the 2023 Clinical and Laboratory Standards Institute guidelines, using 13 antimicrobials of different classes including cefoxitin (FOX, 30 µg), oxacillin (OX, 1 µg), tetracycline (TE, 30 µg), erythromycin (E, 15 µg), azithromycin (AZM, 15 µg), chloramphenicol (C, 30 µg), ciprofloxacin (CIP, 5 µg), trimethoprim/sulfamethoxazole (SXT, 25 µg), gentamycin (CN, 10 µg), rifampin (RA, 5 µg), linezolid (LZD, 30 µg), and clindamycin (DA, 2 µg) sourced from OXOID Ltd. (Hampshire, UK), while vancomycin was determined with minimum inhibitory concentrations (MICs). *S. aureus* ATCC 25,923 was used as a quality control strain for antimicrobial susceptibility testing. The plates were incubated at 37 °C for 18–24 h. After overnight incubation, the zone of inhibition was measured and interpreted as susceptible, intermediate, and resistant based on the recommendation of CLSI (2023) [74]. All antimicrobial susceptibility tests were repeated three times. Multi-drug resistance patterns of the isolates were identified according to the guideline described by Magiorakos et al. [75]. 

The D test method was carried out in accordance with Chavez-Bueno S et al. [76] in order to determine whether or not inducible resistance to clindamycin develops. In Brief, the bacterial isolates were plated on a Mueller–Hinton agar plate at a MacFarland concentration of 0.5 to evenly cover the agar surface. Clindamycin and erythromycin disks, containing 2 μg and 15 μg of each antibiotic, were placed in the middle of the plate separated by a distance of 1.5 cm between the edges. Plates were incubated at 37 °C for 24 h. Inducible resistance to clindamycin was defined as the blunting of the clear circular area of no growth surrounding the clindamycin disk on the side adjacent to the erythromycin disk, and a positive D test result indicated that this type of resistance had been induced. It was determined the D test was negative since there was no evidence of a blunted zone of inhibition, which demonstrates that the strain is in fact susceptible to clindamycin. 

## 5. Conclusions

Through the course of our research, we were able to present a comprehensive analysis that shed light on the proportional distribution of *S. aureus*, methicillin-resistant *staphylococcus aureus* (MRSA), and coagulase-negative staphylococci carrying *mec*A or *mec*C genes in various food categories such as meat, general food items, and pickled foods, as well as in food containers across the rural landscape of northeastern Thailand. These findings bring to light possible concerns with regard to public health, more notably those concerning the environmental contamination of staphylococci that are present within the food chain. It is essential to understand that the existence of these bacteria in meat and other food products may serve as a possible source of antimicrobial resistance and enterotoxin genes, leading to cross-contamination between the population and livestock. As a result, it is necessary to give careful consideration to the control of these bacteria and take appropriate preventative actions in order to limit the risks associated with their presence.

## Figures and Tables

**Table 1:** The number of *mec*A- and *mec*C-positive strains, *S. aureus* and MRSA in different types in this study.

Category of Samples	No. of Sample	No. of Sample Positive for *mecA*	No. of Sample Positive for *mecC*	No. of Sample Positive for *S. aureus*	No. of Sample Positive for MRSA	Enterotoxin Genes *(SEj*, *SEl*, *SEq*, *SEm*, *SEr*)
Food container	80	-	-	-	-	-
Food samples	30	-	-	-	-	-
Pork	30	4 (1.74%)	1 (0.43%)	6 (2.6%)	2 (0.87%)	-
Chicken	30	2 (0.87%)	-	19 (8.26%)	-	2
Beef	30	2 (0.87%)	-	5 (2.17%)	1 (0.43%)	1
Fermented food	30	-	-	2 (0.87%)	-	2
Total	230	8 (3.47%)	1 (0.43%)	32 (13.91%)	3 (1.3%)	5 (2.17%)

**Table 2:** Genetic characteristics and resistance profiles of MRSA and mecA- and mecC-positive isolates.

ID	Sample	mecA/C	Species	SCCmec Types	STs	Spa Types	Resistance Profiles **	
P(2)12.4	Pork	mecA	MRSA	* UN	ST9	t526	FOX-OX-E-DA-CN-AZM -TE-CIP-SXT-C- D+	MDR
B(3)1.2	Beef	mecA	MRSA	IV	ST22	t005	FOX-OX-E-DA-CN-AZM -CIP-D^+^	MDR
B22.5	Beef	mecA	MRSA	V	new	new	FOX-OX -E-CN-AZM-CIP	MDR
P3.5	Pork	mecA	*S. sciuri*	* UN.	-	-	FOX-OX-DA-AZM-TE -SXT	MDR
P(3) 1.2	Pork	mecA	*S. haemolyticus*	* UN.	-	-	FOX-OX-DA-AZM-TE	MDR
P(3) 1.3	Pork	mecA	*S. sciuri*	* UN.	-	-	FOX-OX -DA-TE	MDR
P(3) 1.5	Pork	mecA	*S. haemolyticus*	* UN.	-	-	FOX-OX -DA--TE	MDR
C49.2	Chicken	mecA	*S. haemolyticus*	III	-	-	FOX-OX	-
C49.4	Chicken	mecA	*S. simulans*	* UN.	-	-	FOX-OX -TE	MDR
B57.3	Beef	mecA	*S. warneri*	V	-	-	FOX-OX -E-DA- AZM -TE-C	MDR
B79.1	Beef	mecA	*S. warneri*	V	-	-	FOX-OX -E-DA-AZM-TE	MDR
P20.3	Pork	mecC	*S. xylosus*	* UN.	-	-	OX-TE	-

* UN = unidentified, ** FOX = cefoxitin, OX = oxacillin, TE = tetracycline, DA; clindamycin, E = erythromycin, AZM = azithromycin, C = chloramphenicol, CIP = ciprofloxacin, ST = strain, SXT = trimethoprim/sulfamethoxazole, CN = gentamycin, D^+^ = induce clindamycin resistance. MDR; multidrug-resistant.

**Table 3:** Sequences primers of target genes in the current study.

Target Gene	Primer Sequence (5′-3′)	Size (bp)	Reference
*femA*	F: CGATCCATATTTACATATCA R: ATAACGCTCTTCGTTTAGTT	450	[66]
*mecA*	F: ACGAGTAGATGCTCAATATAA R: CTTAGTTCTTTAGCGATTGC	293	
*Luks*	F: CAGGAGGTAATGGTTCATTT R: ATGTCCAGACATTTTACCTAA	151	
*mecC*	F: GAAAAAAAGGCTTAGAACGCCTC R: GAAGATCTTTTCCGTTTTCAGC	138	[67]
*SEj*	F: CACCAGAACTGTTGTTCTGCTAG R: CTGAATTTTACCATCAAAGGTAC	114	[65]
*SEl*	F: TGGACATAACGGCACTAAAA R: TTGGTARCCCATCATCTCCT	145	
*SEq*	F: ATACCTATTAATCTCTGGGTCAATG R: AATGGAAAGTAATTTTTCCTTTG	222	
*SEm*	F: AGTTTGTGTAAGAAGTCAAGTGTAGA R: ATCTTTAAATTCAGCAGATATTCCATCTAA	178	
*SEr*	F: TCCCATTCCTTATTTAGAATACA R: GGATATTCCAAACACATCTGAC	440	

## Data Availability

Not applicable.
